# The Application of Virtual Reality for Preoperative Planning of Lymphovenous Anastomosis in a Patient with a Complex Lymphatic Malformation

**DOI:** 10.3390/jcm8030371

**Published:** 2019-03-15

**Authors:** Guido Giacalone, Takumi Yamamoto, Florence Belva, Akitatsu Hayashi, Yoav Dori, Menekhem M. Zviman, Mieke Gysen, Hannah H. Nam, Matthew A. Jolley, Motoi Kato

**Affiliations:** 1 Department of Lymphatic Surgery, Sint-Maarten Hospital, 2800 Mechelen, Belgium; Florence_belva@hotmail.com; 2 Department of Plastic and Reconstructive Surgery, National Center for Global Health and Medicine (NCGM), Tokyo 162-8655, Japan; vasko3rikov1meister918@yahoo.co.jp; 3 Department of Breast Center, Kameda Medical Center, Chiba 296-8602, Japan; promise6me5now@gmail.com; 4 Center for Lymphatic Imaging and Interventions, Children’s Hospital of Philadelphia/Hospital of the University of Pennsylvania, Philadelphia, PA 19104, USA; doriy@email.chop.edu; 5 Department of Radiology, Perelman School of Medicine of the University of Pennsylvania, Philadelphia, PA 19104, USA; zvimanm@email.chop.edu; 6 Department of Nuclear Medicine, Heilig Hartziekenhuis, 2400 Mol, Belgium; mieke.gysen@azmol.be; 7 Department of Anesthesiology and Critical Care Medicine, Children’s Hospital of Philadelphia, Philadelphia, PA 19104, USA; namh@email.chop.edu (H.H.N.); jolleym@email.chop.edu (M.A.J.); 8 Division of Pediatric Cardiology, Children’s Hospital of Philadelphia, PA 19104, USA; 9 Saitama Children’s Medical Center, Department of Plastic and Reconstructive Surgery/Lymph Clinic, Saitama 330-8777, Japan; motoikato25@gmail.com

**Keywords:** virtual reality, lymphovenous anastomosis, lymphatic malformation, lymphedema

## Abstract

The management of lymphatic malformations (LMs) is challenging, particularly for large and complex lesions involving anatomical structures in the adjacent tissue. While lymphovenous anastomosis (LVA) has been reported as an effective treatment for lymphedema, it has hardly been described as a treatment for LM. Virtual reality has the ability to visualize human structures in three dimensions and can be used for the preoperative planning of complex cases. Here, we describe the first case of the management of an LM by LVA preoperatively planned with virtual reality. A young woman presented with an LM previously treated by gross excision. Following persistent complaints of swelling, a minimally invasive microsurgical intervention was planned. The results of the single photon emission tomography with computed tomography (SPECT-CT) and lymphoscintigraphy were analyzed using a virtual reality program, and a 3D patient-specific model was constructed. Based on the combined findings of this 3D model and lymphography with a fluorescent marker, a precise skin incision could be determined and one lymph vessel was anastomosed to a nearby vein. The swelling of the thigh reduced and the discomfort disappeared. Although more reports are needed to confirm its efficacy, LVA planned with virtual reality constructed images appears to be a valuable treatment option for complex lesions, including LMs.

## 1. Introduction

Lymphatic malformations, also known as lymphangiomas, are a complex group of congenital vascular anomalies that do not undergo involution and can cause pain, swelling, and functional impairment [1,2]. Given the lack of treatment protocols, their management currently depends mainly on the clinical presentation [3]. Also, the indications for surgical interventions are not uniformly defined. Treatment of lymphatic malformations by resection of the lesion not only causes iatrogenic damage to the surrounding tissue but is mostly incomplete, resulting in persistent morbidity throughout life [2]. Lymphedema may develop after any trauma to lymphatics, especially large surgical resections and those involving lymph node excisions. While lymphovenous anastomosis (LVA) is an established and minimally invasive surgical treatment option for peripheral lymphedema [4], reports of microsurgical treatment for lymphatic anomalies are scarce [5,6]. It is, however, reasonable to assume that a bypass between the lymphatic malformation and a vein will decrease the intra-lesion inflow and hence the size of the cystic lesion, with subsequent volume reduction of the limb. However, lymphatic malformations often distort normal anatomy and extend to deep structures, making surgical planning and treatment challenging.

Virtual reality is an emerging field with the ability to visualize and interact with complex human structures in three dimensions. Particularly in the field of medicine, virtual reality applications are increasingly used for the teaching and training of students [7] and surgeons [8]. Moreover, virtual reality constructed images can serve as tools for a patient-specific simulation of a planned surgical intervention, especially in cases with complicated anatomy.

Here, we describe the first case of microsurgical treatment of a lymphatic malformation preoperatively planned with virtual reality.

## 2. Case Report

### 2.1. Clinical Aspects

**Figure 1:**
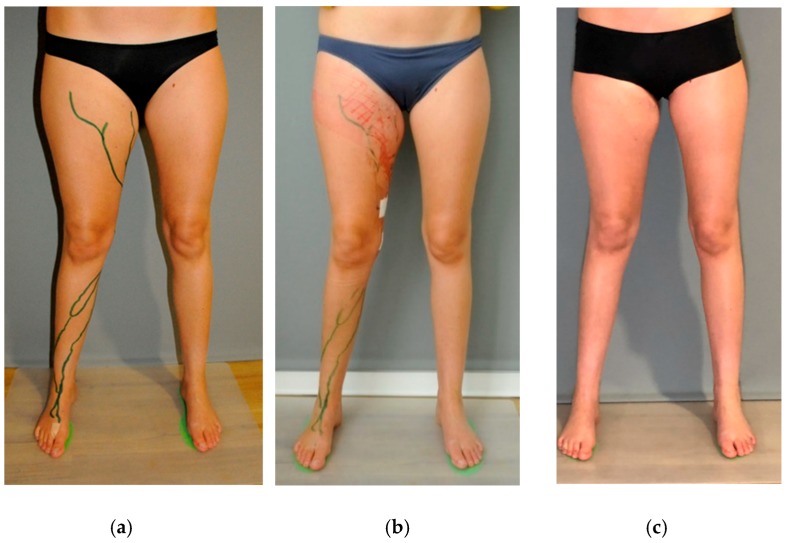
A young woman with a lymphatic malformation at the right thigh. (**a**) Preoperative status (**b**) Immediate postoperative status. Note the considerable decrease in volume of the right thigh. (**c**) Clinical outcome 4 months post-operation.

A 27-year-old female patient with a simple vascular malformation—described as ‘lymphangioma’ [9]—at the level of the right thigh and groin was referred for treatment (Figure 1a). The lesion had been present since birth and had been partially resected at the age of 15 years. The large, but incomplete, excision had resulted in lymphedema of the right leg and thigh. At the time of referral, there were ongoing complaints of swelling of the right limb and a feeling of tension, particularly at the level of the groin. The volume difference between the affected and unaffected leg was 1222 mL, measured by an optoelectronic limb volumeter (Perometer, Pero-System, Wuppertal, Germany) [10]. 

Conservative treatment by means of manual lymph drainage and compression garments was initiated; however, results were unsatisfactory. Given the refractory swelling and continued complaints of a feeling of pressure at the level of the groin, and given the applied gross excision in the past, a minimally invasive surgical intervention was planned. 

Lymphatic mapping was performed by means of lymphography (PDE Neo II, Hamamatsu, Japan), with the fluorescent marker indocyanine green (ICG). Patent lymphatics at the medial side of the thigh, visualized by near-infrared technology, were marked on the skin [11]. 

**Figure 2:**
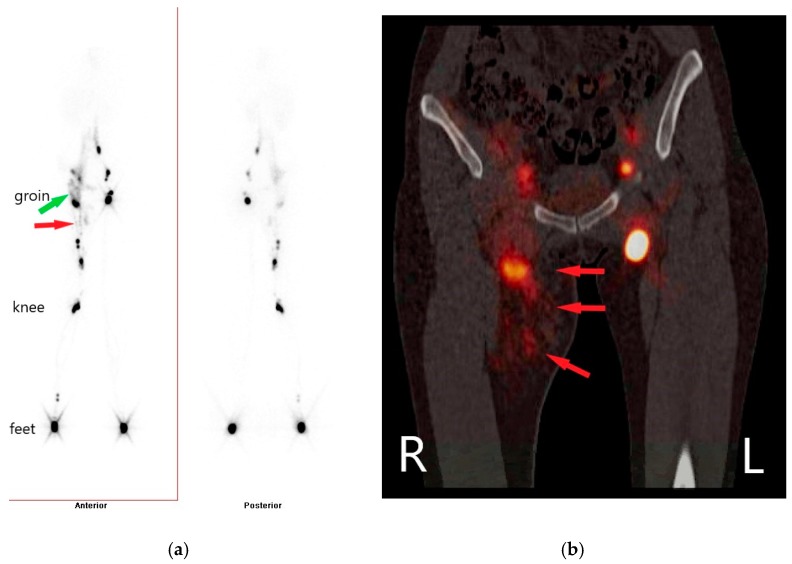
(**a**) Lymphoscintigraphy: uptake of radio-colloid tracer at the level of the right thigh (red arrow) and extending into the abdomen (green arrow). (**b**) Hybrid images of single photon emission tomography and computed tomography (SPECT-CT) and lymphoscintigraphy: visualization of radio-colloid tracer at the level of the right thigh pointing to the presence of lymphatic structures (red arrows).

A lymphoscintigraphy image of the lower extremities showed a pronounced uptake of radioactive tracer on the medial side of the thigh (Figure 2a). The combined examination of the single photon emission tomography (SPECT) with computed tomography (CT) in a hybrid system (SPECT-CT and lymphoscintigraphy) revealed an accumulation of radio-colloid tracer along the course of lymphatics at the medial side of the thigh, pointing to either lymph nodes, a lymphocele, or a lymphatic malformation (Figure 2b).

**Figure 3:**
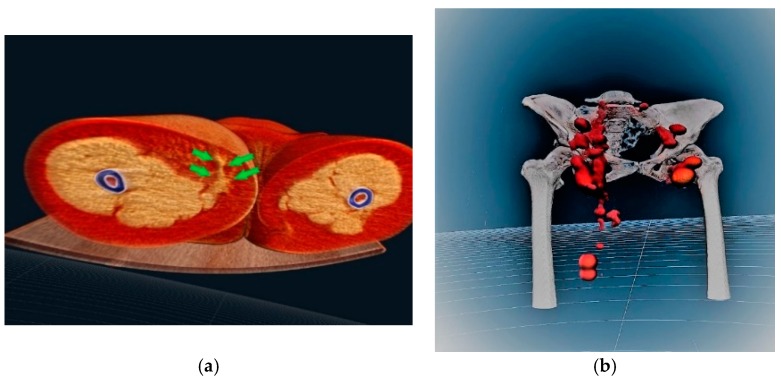
(**a**) 3D constructed model based on the fusion of the radiographic images and obtained by virtual reality software showing the lymphatic malformation (green arrows) (**b**) 3D reconstructed hybrid image of the SPECT-CT and lymphoscintigraphy obtained by virtual reality software: visualization of the lymphatic structures of the lower limbs. Note the extension of the malformation (conglomerate of tracer) from the region of the right thigh up to the abdominal cavity.

Finally, the images from the SPECT-CT and lymphoscintigraphy were analyzed with a virtual reality program (Medicalholodeck, Zurich, Switzerland) [12] that allowed the visualization in a 3D model of the localization of the lymphatic malformation (Figure 3a, Video S1), with its extension into the abdominal cavity, in a virtual reality environment (Figure 3b, Video S2).

**Figure 4:**
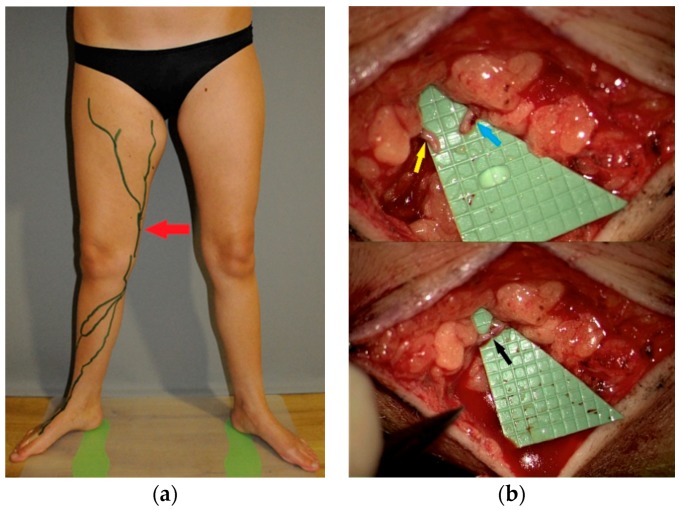
(**a**) Patent lymphatic vessels identified by indocyanine green (ICG) lymphography are marked on the skin. The site of incision is marked by an arrow. (**b**) Exposure of the lymphatic vessel (yellow arrow) and the vein (blue arrow) with diameters of 0.5 mm and 0.6 mm, respectively, through a 3 cm skin incision (higher panel) prior to the lymphovenous anastomosis (black arrow, lower panel).

Based on the 3D model and the mapping by ICG lymphography, the incision site was determined (Figure 4a), and an end-to-end anastomosis between a patent lymphatic vessel (diameter 0.5 mm) and an adjacent vein (diameter 0.6 mm) was performed using an 11/0 suture (Figure 4b). The patency of the lymphovenous anastomosis was confirmed intraoperatively by ICG lymphography. Postoperative compressive bandaging was applied. The procedure was performed under loco-regional anesthesia.

The postoperative course was uneventful. Immediately after surgery, the feeling of tension disappeared and the volume of the right thigh decreased (Figure 1b). Four months postoperative, the result was very good, with further reduction of the volume of the right leg; the difference in volume between the left and right leg was reduced to 224 mL (Perometer 400 NT) [10] (Figure 1c).

### 2.2. Technical Aspects

The conventional images of the SPECT-CT and lymphoscintigraphy, stored as DICOM data, were processed by dedicated software [12]. The DICOM viewer of Medicalholodeck allowed us to handle the DICOM data and merge them into a single virtual reality model. In order to visualize this 3D patient-specific model, a virtual reality headset (Vive Pro, HTC corporation) and a computer with a powerful graphics card (GTX-1080ti) and a large memory (32 GB RAM) were needed. The result was a detailed 3D image of the anatomical structures and, more precisely, of the lymphatic malformation in a virtual reality environment (Figure 3a,b, Videos S1 and S2).

The treatment was conducted in accordance with the Declaration of Helsinki and under the institutional ethical review board of the hospital. Written informed consent was obtained. The patient provided written informed consent for the publication and the use of the images.

## 3. Discussion

This report describes the first case of lymphovenous anastomosis planned using virtual reality and successfully applied to a patient with a lymphatic malformation.

Lymphovenous anastomosis as a treatment for lymphatic malformations has rarely been described in literature. Kato et al. [5] successfully created a bypass between the lymphatic malformation and a vein in a young patient with Klippel–Trenaunay Syndrome. The clinical outcome of an orbital micro-cystic lymphatic malformation in a child also improved dramatically after LVA treatment [6]. Although the approach of deviating the lymph flow by means of a bypass is physiological, the complex anatomy and the relations of normal and abnormal anatomical structures are a serious impediment to a more widespread use of the technique.

While virtual and augmented reality-based navigation surgery is commonly applied in neurosurgery and maxilla-facial surgery [13], its application to lymphatic surgery has not been previously described. However, we believe that virtual reality will be particularly useful for understanding the complex anatomy of vascular and lymphatic abnormalities. The complex anatomy of the lesions, as well as iatrogenic scarring as a result of previous surgeries, necessitates a comprehensive preoperative understanding to define the optimal and least invasive surgical approach. Indeed, in our case there was a subjectively high correlation between the 3D constructed images and the perioperative findings, making execution of the surgical plan easier.

The strategy of the intervention was to identify the lymphatics leaking into the malformation and to interrupt the inflow towards the lesion by redirecting the lymph towards the venous system. To do so, it was mandatory to find the exact localization of the afferent lymphatic vessels. Lymphoscintigraphy is the gold standard imaging modality of lymphatic disorders, but it provides only static information [14]. SPECT-CT is of particular value because conventional planar imaging is not able to discriminate between lymph nodes and other structures, including lymphangioma; however, the hybrid examination does not add to the spatial information of the lymphatic structures in relation to the surrounding tissue. Therefore, the conversion of cross-sectional radiological images into 3D reconstruction and the generation of 3D visualization in virtual reality was required. This enabled us not only to see in 3D the lymphatic drainage pathways and the localization of the lymphatic malformation, but also to plan and perform the intervention without iatrogenic damage to the adjacent structures. This was an important goal, as previous resection was only temporarily successful, and scarring is a well-known complication of multistage surgery.

The advantages of virtual reality in this setting are obvious. Firstly, the visualization of complex human structures in 3D with a head-mounted display provides a realistic representation of the human body, which is a three-dimensional object. Looking at it in its original dimensions in an immersive virtual reality environment, rather than on an artificial two-dimensional image on a screen, enables the viewer to gain insight into the actual anatomy of the patient. As such, patients with a complex lymphocele are ideal candidates for treatment with virtual reality guided LVA. Secondly, the patient-specific model visualized in a 3D virtual space can be simultaneously viewed by others. Moreover, one can collaborate on the medical DICOM data using a dedicated platform provided by the software (The Medicalholodeck Cloud). Although we did not use this platform in our case, it could be useful for discussing the approach of complicated cases with other surgeons around the world. Furthermore, since several care providers can collaborate and communicate in real time thanks to this Cloud, difficulties can be discovered before the actual surgical intervention is carried out. 

However, virtual reality devices and associated hardware and software remain relatively expensive and their use is time-consuming and difficult to integrate into standard practice. In our case, whilst only 15 min were required to study the anatomy of the lymphatic malformation in order to plan the subsequent intervention, one must account for at least 1 hour of preparation for the data to be processed by the dedicated software. On the other hand, as a result of the rapid progress in this field of technology, costs associated with hard- and software are expected to decrease. As it is highly likely that virtual and augmented reality will become more popular in all fields of medicine in the near future, medical staff will easily gain experience in handling the medical DICOM data of the patient.

In conclusion, we successfully treated a lymphatic malformation after preoperative planning using the visualization of standard imaging in virtual reality. Although further development is needed, our initial experience suggests the utility of virtual reality for the planning of surgical interventions on complex lymphatic lesions. 

## Supplementary Materials

The following are available online at https://www.mdpi.com/2077-0383/8/3/371/s1, Video S1: Visualization of the preoperative status based on radiographic images in virtual reality. Video S2: 3D model of the hybrid images of SPECT-CT and lymphoscintigraphy in virtual reality.
